# Novel Injectable Collagen/Glycerol/Pullulan Gel Promotes Osteogenic Differentiation of Mesenchymal Stem Cells and the Repair of Rat Cranial Defects

**DOI:** 10.3390/gels10120775

**Published:** 2024-11-28

**Authors:** Xin Wang, Satoshi Komasa, Yoshiro Tahara, Shihoko Inui, Michiaki Matsumoto, Kenji Maekawa

**Affiliations:** 1 Department of Removable Prosthodontics and Occlusion, Osaka Dental University, 8-1, Kuzuhahanazono-cho, Hirakata-shi 573-1121, Osaka, Japan; celiawx1823@outlook.com (X.W.); inui-s@cc.osaka-dent.ac.jp (S.I.); maekawa-k@cc.osaka-dent.ac.jp (K.M.); 2 Department of Oral Health Sciences, Osaka Dental University, 1-4-4, Makino-honmachi, Hirakata-shi 573-1144, Osaka, Japan; 3 Department of Chemical Engineering and Materials Science, Doshisha University, 1-3 Tatara-Miyakodani, Kyotanabe 610-0321, Kyoto, Japan; ytahara@mail.doshisha.ac.jp (Y.T.); mmatsumo@mail.doshisha.ac.jp (M.M.)

**Keywords:** collagen, pullulan, injectable gel, mechanical properties, osteogenic differentiation, bone repair, irregular bone defect

## Abstract

Bone tissue engineering is a technique that simulates the bone tissue microenvironment by utilizing cells, tissue scaffolds, and growth factors. The collagen hydrogel is a three-dimensional network bionic material that has properties and structures comparable to those of the extracellular matrix (ECM), making it an ideal scaffold and drug delivery system for tissue engineering. The clinical applications of this material are restricted due to its low mechanical strength. In this investigation, a collagen-based gel (atelocollagen/glycerol/pullulan [Col/Gly/Pul] gel) that is moldable and injectable with high adhesive qualities was created by employing a straightforward technique that involved the introduction of Gly and Pul. This study aimed to characterize the internal morphology and chemical composition of the Col/Gly/Pul gel, as well as to verify its osteogenic properties through in vivo and in vitro experiments. When compared to a standard pure Col hydrogel, this material is more adaptable to the complexity of the local environment of bone defects and the apposition of irregularly shaped flaws due to its greater mechanical strength, injectability, and moldability. Overall, the Col/Gly/Pul gel is an implant that shows great potential for the treatment of complex bone defects and the enhancement of bone regeneration.

## 1. Introduction

The skeleton is a dynamic organ that is constantly being remodeled in order to adapt bone structure to shifting mechanical demands and to facilitate the repair of micro-damages in the bone matrix. While bone tissue may sufficiently regenerate to mend minor injuries, it becomes more problematic for bone defects greater than the crucial size (commonly ≥ 2 cm, depending on the anatomical region) to recover unaided [1,2]. Moreover, bone defects usually appear irregular in shape. Therefore, bone grafting and implanted bone-substitute materials that match irregular bone defects have become critical strategies for promoting bone repair [3,4]. However, it has been difficult to adequately fill regions that have uneven bone defects with various prefabricated bone restoration materials. Autogenous bone grafting has been used for centuries and is still the gold standard for the treatment of bone defects [5,6]. However, autologous bone grafting is considerably restricted by many factors, such as the risk of operation, destruction to the donor area (such as infection, bleeding, pain, instability, etc.), inadequate supply of materials, and complications, among others [7,8].

To avoid these risks, hydrogel tissue-engineered substrates have gained notable attention owing to their outstanding characteristics. Hydrogel is a polymer substance with a three-dimensional network structure that can simulate the natural tissue environment and provide a favorable matrix for cell growth and bone regeneration [9,10]. Type I collagen mediates extracellular signals into cells upon interaction with the integrin receptors of cell membranes, which in turn stimulates the osteogenic differentiation of bone marrow-derived mesenchymal stem cells (BMSCs) [11,12]. Moreover, collagen, as a key component of the ECM in bone tissue, is quickly degradable, biocompatible, and may form gels under physiological circumstances. Therefore, collagen-based hydrogels are optimal substances for optimum bone tissue regeneration and repair. However, due to the lack of mechanical strength and quick breakdown of collagen fibers by collagenase, collagen gels are severely restricted in bone tissue engineering applications. One technique of enhancement is to introduce extra-polymeric elements to boost the strength and durability of collagen gels [13,14].

The ECM provides structural support for cells and also regulates cell differentiation [15]. The elasticity or stiffness of the ECM impacts key cellular activities, including attachment, spreading, growth, migration, differentiation, and organoid creation. Mechanistically, the current understanding is that cells connect to the substrate via integrins and exert traction by using actin contractile forces. These forces perceive changes in substrate stiffness via integrins, conformational changes in mechanosensitive proteins, and activation of mechanosensitive ion channels. These modifications may in turn impact long-term cellular processes, such as differentiation, via persistent perception, mechanistic memory, and epigenomic changes [16]. Therefore, viscoelasticity and stiffness strength of the gel is also an essential consideration for gel design. The highly porous structure of biomaterials provides space for cell growth, migration, and nutrient exchange [17,18]. Therefore, suitable gels should introduce porous structures of different sizes to meet the different requirements needed for cell growth and differentiation.

Atelocollagen (Col), obtained by eliminating the N- and C-terminal telopeptide sections, is a highly promising biomaterial due to its good biocompatibility and diminished immunogenicity [19]. Kim et al. predicted that Col triggers the production of Sox9, a pivotal factor for chondrogenic differentiation [20]. Studies have shown that Col gel can be used alone or in combination with bone marrow aspirate concentrate to promote bone tissue repair and regeneration [21,22].

Glycerol (Gly) is a cost-effective and easily available polyhydroxylated material. The strong hydrogen bonding between Gly and water molecules hinders water evaporation, which gives Gly-modified gels strong water-holding properties [23,24]. Several studies have revealed that following Gly modification, the mechanical characteristics of various gels were increased and that they have the possibility to be employed in soft tissue engineering [25,26].

Pullulan (Pul), a polysaccharide polymer consisting of maltotriose units, is biodegradable, with numerous benefits comprising non-toxicity and non-immunogenicity [27]. Currently, little research has been carried out on Pul gels for bone tissue engineering applications; however, it can have important applications in tissue engineering owing to its distinctive glycosidic linkage and facile chemical derivatization on hydroxyl groups in its main chain [28]. Fricain et al. reported that Pul composite scaffolds induced a highly mineralized tissue in three separate bone locations in goats [29].

In the present study, we demonstrated a novel Col gel modified with Gly and Pul, which was constructed via freeze-drying. The most essential benefits of freeze-dried gels are their comparatively simple storage and extended shelf-life. Initially, the gel does not contain any water; however, after absorbing water, it can transform into a hydrogel that is evenly porous. Compared to traditional pure collagen hydrogels, the Col/Gly/Pul gel has been observed to have moldability, injectability, enhanced mechanical strength, and resistance to degradation. The viscoelasticity and pore size properties of the Col/Gly/Pul gel were analyzed, and its effects on behaviors of rat BMSCs (rBMSCs) and abilities to support the healing of rat cranial bone defects were also explored via in vitro and in vivo experiments. Overall, this research presents a novel strategy for the treatment of clinical bone defects.

## 2. Results and Discussion

### 2.1. FT-IR, Thixotropic Study and Swelling Behavior of the Col/Gly/Pul Gel

**Figure 1:**
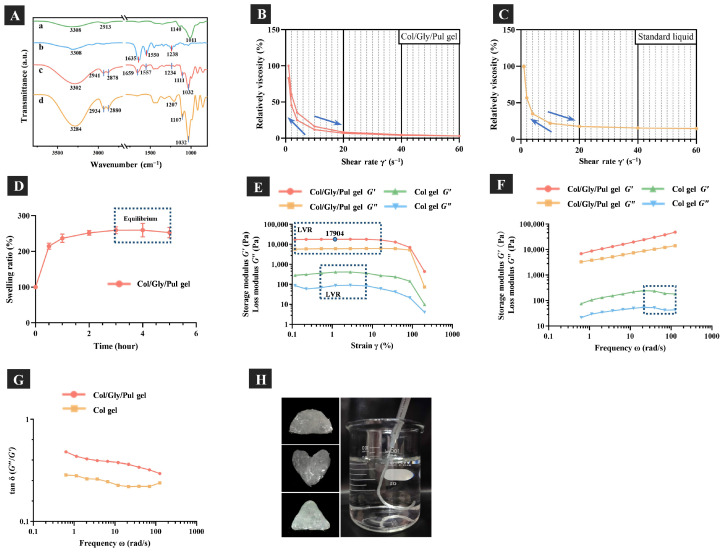
(**A**) Fourier-transform infrared spectra: (a) pullulan (Pul), (b) atelocollagen (Col), (c) Col/Gly/Pul gel, and (d) glycerol (Gly). (**B**,**C**) Thixotropic studies of Col/Gly/Pul gel and standard liquid. (**D**) Swelling behavior of the Col/Gly/Pul gel. (**E**) Amplitude-strain, (**F**) frequency, and (**G**) tan δ (*G*″/*G*′) of frequency sweeps. LVR, linear viscoelastic region. (**H**) Images showing the moldability and injectability of the Col/Gly/Pul gel.

The Fourier-transform infrared (FT-IR) spectra (Figure 1A) demonstrated characteristic peaks of the Col/Gly/Pul gel and its raw materials. The characteristic peaks of Pul are displayed in Figure 1A(a) [30]. The C-H stretching vibrations of Gly were responsible for the absorption bands obtained at 2878, 2880, 2934, and 2941 cm^−1^, and its C-O stretching vibrations observed at 1032, 1107, and 1111 cm^−1^ [31]. A variety of distinctive peaks are produced by the amide groups that are found in the collagen backbone. In particular, the amide I band (1635, 1659 cm^−1^) forms mainly due to C=O stretching vibrations. The amide Ⅱ band of the N-H bending vibrations could be observed at 1550 and 1557 cm^−1^. The amide Ⅲ band of the C=O stretching vibrations was observed at 1234 and 1238 cm^−1^ [32,33]. The characteristic peaks of Gly and Col were presented in the Col/Gly/Pul gel. As no other distinctive peaks were observed, this indicates that the chemical structure of Col was not disrupted, and that Col, Pul, and Gly physically wrapped around each other to form the gel. The Col/Gly/Pul gel can thus be classified as a physically cross-linked gel [34]. As compared to a chemically cross-linked gel, the interaction of a polymer network in a physically cross-linked gel like a collagen hydrogel is relatively weak, and an internal polymer chain can be reversible [35]. This flexible network structure is suitable for tissue engineering that needs deformable cell adhesion scaffolds.

The results of thixotropic studies are shown in Figure 1B,C. Compared with non-thixotropic standard liquid, the Col/Gly/Pul gel is thixotropic. This means that it changes from a solid to a liquid when squeezed by an external force and can be injected. As shown in Figure 1D, Col/Gly/Pul gel absorbed a large amount of water within 30 min, and it reached equilibrium at 3 h. The swelling behavior suggests that Col/Gly/Pul gel is very hydrophilic, probably due to the high content of glycerol.

### 2.2. Viscoelasticity of Materials

The linear viscoelastic region (LVR) of gels was obtained via an amplitude-strain sweep at a fixed angular frequency of 1 Hz, as shown in Figure 1E. It is important to note that the storage modulus, *G*′, reflects the elastic component of the gel, whereas the loss modulus, *G*″, indicates the viscous component of the gel. In the range of the LVR (Col/Gly/Pul gel: γ1 = 0.12~16.21%; Col gel: γ2 = 0.53~7.08%), both gels maintained stable forms and appropriate extensibilities. The *G*′ and *G*″ of the Col/Gly/Pul gel were, however, significantly higher than those of the Col gel (*p* < 0.05). This indicates that the mechanical strength and adhesion of the Col/Gly/Pul gel were likely enhanced by the doping of Gly and Pul, and that it has excellent elastic properties and deformation resistance [36,37]. In addition, the frequency sweep tests (Figure 1F) showed a consistent upward trend in the *G*′ of the Col/Gly/Pul gel, which was always larger than the *G*″ of the gel. Both the *G*′ and *G*″ of the Col gel began to decrease in the latter part of the external force (shown in the dotted frame), despite the fact that the *G*′ of the Col gel was always higher than the *G*″. This indicates that after a longer period of external force, the Col gel shows a tendency to transform similar to that of a liquid. Meanwhile, the results of *G*′ and *G*″ of the Col/Gly/Pul gel and Col gel were frequency-dependent. As shown in Figure 1G, the values of tan δ for Col/Gly/Pul gel were greater than those for Col gel, and both were greater than 0.1. In the literature [34,38], the frequency dependence of the modulus and the value of tan δ (=*G*″/*G*′ > 0.1) are characteristic of weak gels. Under significant strain, the weak gels’ systems exhibited deformation without rupture, but markedly strong gels, which are strong and brittle without shape-shifting, tend to be more disrupted. This means that, compared with Col gel, Col/Gly/Pul gel has higher strength and better fluidity to accommodate extrusion by external forces. A previous study reported that chemically cross-linked Pul gels were not deformable and behaved as frequency-independent solid-like materials [39,40]. When compared to the previous chemically cross-linked Pul gels, the Col/Gly/Pul gel (a physically cross-linked gel) is a deformable weak gel, which can be better adapted to irregular defects.

**Figure 2:**
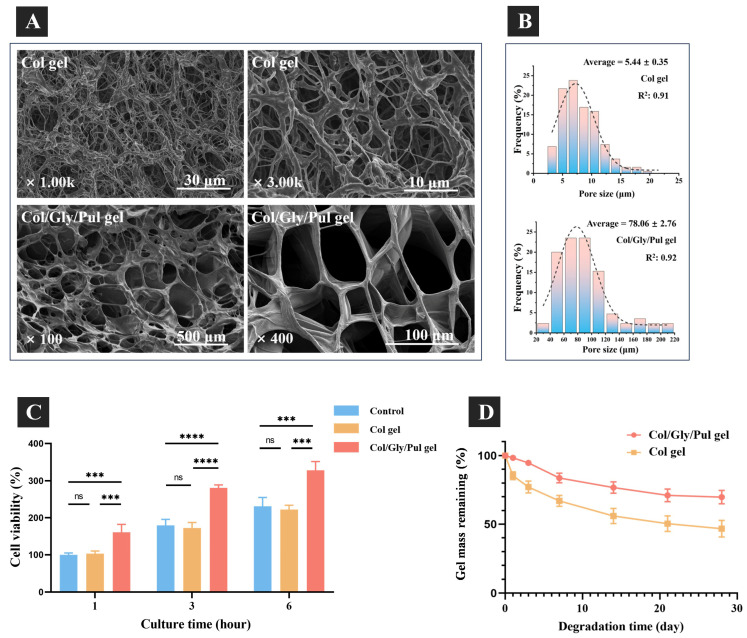
(**A**) Scanning electron micrographs, and (**B**) pore size distribution. (**C**) The cell viability of rBMSCs initial adherent to the control, Col gel, and Col/Gly/Pul gel. Not significant (ns): *p* ≥ 0.05; ***: *p* < 0.001; ****: *p* < 0.0001. (**D**) In vitro degradation of the Col/Gly/Pul gel and Col gel.

Previous research indicated that mesenchymal stem cells (MSCs) largely underwent adipogenic differentiation at an initial modulus of 2.5–5 kPa and predominantly osteogenic differentiation at an initial modulus of 11–30 kPa [41]. With a quickly relaxing gel, which has an initial elastic modulus of 17 kPa, Chaudhuri et al. established that MSCs are capable of producing a mineralized matrix that is rich in collagen I, similar to bone [16]. This is likely because mechanical stimulation of MSCs by the stiffer matrix stimulates RhoA signaling, which promotes osteogenesis and inhibits chondrogenesis [42]. The amplitude-strain sweep tests showed that under a wide range of applied strains or stresses, the Col/Gly/Pul gel was able to maintain a storage modulus (elastic modulus) of 17 kPa. This suggests that the Col/Gly/Pul gel has a relatively suitable initial elastic modulus and the potential to promote MSC differentiation. Moreover, a much higher number of cells initially attached to the surface of Col/Gly/Pul gels compared to that of the control group and Col gels (Figure 2C), not only due to the enhanced viscosity of the Col/Gly/Pul gel itself, but also possibly due to the reduction in plasma myosin-dependent traction in the softer Col gel, which reduced cell adhesion and weakened actin pull, as well as reduced ECM-integrin-cytoskeletal connections [43]. However, on the stiffer Col/Gly/Pul gel, integrin affinity increased, which allows for an increase in adhesive plaque proteins and thus adhesion. But we have not yet explored the dynamic changes of its viscoelasticity in vivo and need to discuss this in future experiments.

### 2.3. Pore Size and Scaffolding of Materials

Figure 2A displays, at varying magnifications, images of the morphology of the two freeze-dried gels that were used in this experiment. Scanning electron microscopy (SEM) analysis showed that both Col/Gly/Pul and Col gels have an obvious three-dimensional network structure and uniform pores. The micrographs also show that pores of the Col/Gly/Pul gel are much more neatly shaped, mostly round or oval-shaped, and that the pore wall is thicker with a stronger connection. The fibers of the Col gel, however, are thin and the crosslinks more disordered. Pore size distribution histograms (Figure 2B) of the gels were plotted in Origin using processed SEM micrographs from ImageJ. The pore size distribution ranged from 2.5~22.5 μm and from 20.00~220.00 μm, and the average pore sizes were 5.44 ± 0.35 μm and 78.06 ± 2.76 μm, for the Col and Col/Gly/Pul gels, respectively. The Col gel had a concentrated micropore size limited to 23 µm; the Col/Gly/Pul gel covered a wide range of pore sizes, from micro- to macropores. The initial adherent cell viability (Figure 2C) indicated that, after cell seeding, the Col/Gly/Pul gel surface had the maximum number of adherent cells at 1, 3, and 6 h among these groups; additionally, the Col gel exhibited identical numbers of adherent cells as in the control group (24-well plate).

**Figure 3:**
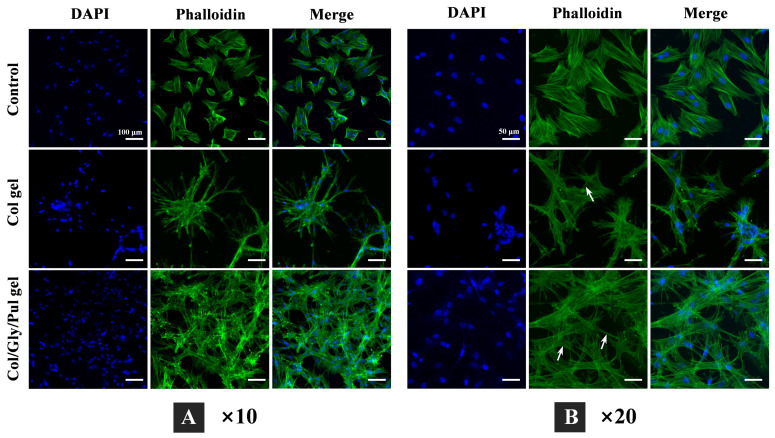
Morphology of rBMSCs attached to the control, Col gel, and Col/Gly/Pul gel after 24 h of incubation. Nuclei were labeled with DAPI (blue), and actin filaments stained with Phalloidin (green). Images obtained at a lower magnification (**A**) show the number and distribution of cells (scale bars = 100 µm), whereas those obtained at a higher magnification (**B**) show the connections among cells attached to the different samples (white arrows indicate interactions among cells; scale bars = 50 µm).

The Col/Gly/Pul gel presented the highest initial cell adhesion. The fluorescence staining findings (Figure 3) of the cells after 24 h of cell attachment revealed that the number of cells connected to the control group was fewer than that of both gels, with a flat morphology and evenly dispersed cells. The quantity of cells that adhered to the Col gel was lower than that of the Col/Gly/Pul gel and closer to that of the control group, and the cells were agglomerated and unevenly distributed, with a three-dimensional elongated morphology and filamentous pseudopods connected to neighboring cells. The Col/Gly/Pul gel contained the maximum number of cells attached to the surface and was uniformly distributed, where the morphology of the cells showed a better extension and three-dimensionality, with sufficiently extended filamentous pseudopods.

The pore size of a gel is a significant component that influences the migration of cells from natural bone tissue or in hydrogels. Microporosity might enhance the surface area and roughness of the gels and could offer additional protein adsorption sites and cellular attachments to induce osteogenesis [44]. Medium pore sizes, such as 10 µm, may improve bone-like apatite production, the exchange rate of inorganic ions, and absorption of bone-forming proteins. Large pore sizes above 100 µm enhance cell migration and translation [9]. A too-small pore size not only limits cell migration and proliferation, but will also limit nutrient and waste exchange in the center of the scaffold [45]. Col/Gly/Pul gels with 20~220 µm pore sizes provide the different-sized spaces required for cell growth. Col gels with a pore size of 2~20 µm may restrict cell migration and spreading. We hypothesize that the incorporation of Gly and Pul enhanced the mechanical strength viscosity, pore size, and wall thickness of the gel. These modifications created an environment that was conducive to cell attachment, proliferation, spreading, and nutrient diffusion. The initial attachment of cells onto the surface of the Col/Gly/Pul gel was well distributed with sufficiently extended filamentous pseudopods, which further facilitated the deep invasion of cells and generation of bone tissue at a later stage.

The cytoskeleton, which is the basis for the maintenance of cellular morphology and for the participation of cells in a variety of important biological activities, is composed of three main structures: microfilaments, microtubules, and intermediate filaments [46]. Actin is the main component of microfilaments and exists in two forms in vivo: globular actin (G-actin,) and fibrillar actin (F-actin). Figure 3 represents cells grown on harder Col/Gly/Pul gel with more abundant and multidirectional stretching of F-actin compared to that in the control and Col gel. This may be due to the fact that physical and mechanical signals from the outer matrix are mediated by protein molecules on the surface of the cell membrane (such as integrins and cadherin). These protein molecules are then transduced intracellularly to regulate the reorganization of assemblies of the skeletal protein molecules mentioned above, and ultimately the reorganization of cytoskeletal microfilaments and microtubules, which results in the maintenance of cellular morphology [47,48]. This is consistent with previous reports. Stem cells cultivated on a hard substrate demonstrated increased spreading, formed more stress fibers, and had a higher growth rate [49]. Rigid substrates caused cells to spread broadly with a polygonal form, similar to that under natural conditions [50]. The cell adhesion (Figure 2C) and morphology (Figure 3) results obtained support these findings.

### 2.4. Degradation Rate of Materials

The degrading behavior of gels was also examined (Figure 2D). The Col/Gly/Pul gel showed no considerable change in mass on days 1–3, degraded at an accelerated rate on days 4–7, but then displayed a decreased degradation rate as the gel continued to hydrolyze slowly in week 2. At the end of week 4, the Col/Gly/Pul gel lost approximately 30% of its mass. The Col gel consistently degraded at a high rate for the first two weeks and lost approximately 40% of its mass; however, the rate of degradation slowed during weeks 3–4, whereafter the gel eventually lost 50% of its mass. Overall, the Col/Gly/Pul gel degraded at a more constant and slower rate.

The ideal scaffold for bone tissue engineering is not a permanent implant and should have integrity in the early stage, providing mechanical support for bone defects and attachment sites for osteoblasts and osteoclasts. In the later stage, the scaffold should be able to degrade or metabolize, leaving enough room for the growth of newly created bone tissue [51]. Eventually, the scaffold may be totally replaced by the newly created bone tissue, restoring the bone deficiency to its regular physiological function. In vitro degradation experiments showed that the initial degradation rate of the Col/Gly/Pul gel was considerably slow, which might provide more attachment sites during initial cell attachment and help maintain a steady and continuous degradation rate. Further, its degradation was stabilized in the mid-to-late stages, whereas that of the Col gel proceeded more rapidly. This suggests that the Col/Gly/Pul gel can provide more attachment sites for cell attachment over a longer period; however, rapid degradation of the Col gel may not be conducive to long-term cell attachment and proliferation. Given that the regeneration of a bone defect typically involves the spreading and proliferation of new bone tissue, the optimal biomaterial degradation rate should coincide with the pace of physiological repair [52]. How to tailor the pace of scaffold deterioration to the replenishment capability of the host tissue is one of the primary issues that remain [53]. However, it is currently unclear how rapidly Col/Gly/Pul gel is degraded in vivo. In the future, more in vivo degradation experiments should be carried out.

### 2.5. In Vitro and Vivo Evaluation of Bone Formation

**Figure 4:**
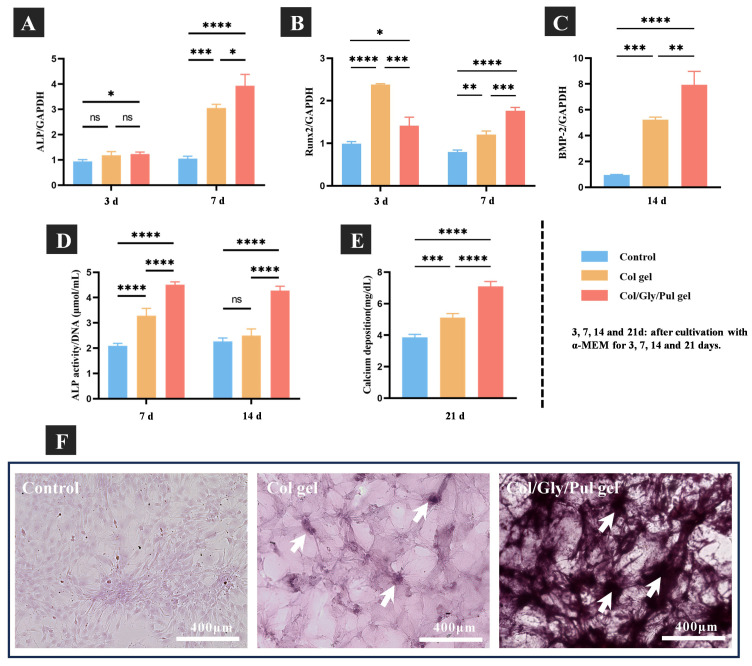
(**A**–**C**) The real-time PCR analysis of specific osteogenesis-related gene expression on the control, Col gel, and Col/Gly/Pul gel. (**D**) Alkaline phosphatase (ALP) activity and (**E**) calcium deposition on the control, Col gel, and Col/Gly/Pul gel. Not significant (ns): *p* ≥ 0.05; *: *p* < 0.05; **: *p* < 0.01; ***: *p* < 0.001; ****: *p* < 0.0001. (**F**) Alizarin Red staining of the control, Col gel, and Col/Gly/Pul gel (white arrows indicate bone nodules).

The specific expression levels of osteogenesis-related genes, concluding those encoding alkaline phosphatase (ALP), runt-related transcription factor 2 (Runx2) and bone morphogenetic protein-2 (BMP-2), were measured using TaqMan real-time quantitative PCR. ALP, as an early indicator of bone formation, plays a crucial role in promoting the differentiation of BMSCs into bone cells [54]. In addition, the transcription factor, Runx2, which belongs to the runt homology structural domain family, is also necessary for osteoblast differentiation [55]. BMP-2, a member of the TGFB (transforming growth factor) superfamily, induces bone formation and regeneration [56]. At three days after osteogenic induction, ALP gene expression was higher in cells of the Col/Gly/Pul gel than in the control group and Col gel. Although no statistically significant differences between the control group and Col gel were measured, ALP expression tended to be higher in the Col gel. Runx2 expression was highest in the Col gel and higher in the Col/Gly/Pul gel than in the control. After seven days of culture, ALP and Runx2 expression levels were significantly higher in the Col/Gly/Pul gel than in the Col gel and the control group (*p* < 0.05). Compared to the Col gel and the control group, the cells cultured on the Col/Gly/Pul gel revealed greater levels of BMP-2 mRNA after 14 days of culture (Figure 4A–C).

Figure 4D illustrates the quantitative ALP activity results. The relative ALP activity of cells on the Col/Gly/Pul gel was considerably greater than that of cells on the Col gel and control after 7 and 14 days. No significant differences between the control group and Col gel were observed on day 7. Calcium deposition, a late marker of ECM mineralization, was quantified for each group after 21 days of differentiation. As shown in Figure 4E, the greatest level of calcium deposition was detected in the Col/Gly/Pul gel, and that on the Col gel was much greater than that on the control.

In Figure 4F, Alizarin Red staining after 28 days of culture showed an even distribution of cells on the control, but few distinct areas of staining. There were some limited areas of staining on the Col gel, indicating some degree of mineralization and a rise in the number of bone nodules compared to that in the control group, but still not by a significant amount. There were distinct and widely distributed areas of dark staining on the Col/Gly/Pul gel and a considerable increase in the number of bone nodules, suggesting an elevated level of mineralization. In conclusion, the control group showed almost no mineralization, the Col gel showed some mineralization, and the Col/Gly/Pul gel group showed the most notable mineralization with the highest number of bone nodules and most pronounced staining. These findings demonstrate the greater capacity of the Col/Gly/Pul gel to consistently promote osteointegration.

**Figure 5:**
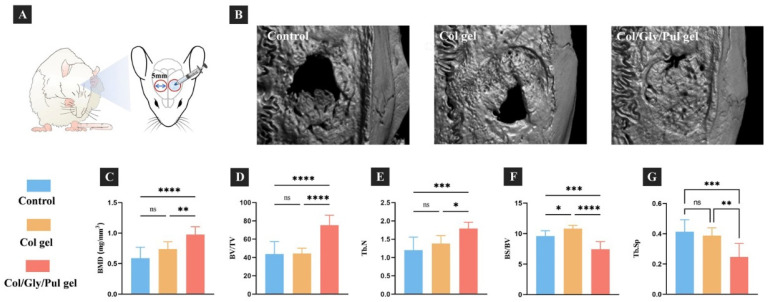
Effect of the Col/Gly/Pul gel on bone formation in a rat cranial defect model. (**A**) Treatment protocol for cranial bone defects in rats. Briefly, after separating the periosteum, a 5 mm cylinder-shaped bone defect was prepared on both sides of the sagittal midline of the rat parietal bone and then treated with or without gel. (**B**) Reconstructed three-dimensional micro-CT images of bone tissues after eight weeks. (**C**) Bone mineral density (BMD), (**D**) bone volume-to-total volume ratio (BV/TV), (**E**) trabecular number (Tb. N), (**F**) bone surface-to-bone volume ratio (BS/BV), and (**G**) trabecular separation (Tb. Sp) around the control, Col gel, and Col/Gly/Pul gel after eight weeks. Not significant (ns): *p* ≥ 0.05; *: *p* < 0.05; **: *p* < 0.01; ***: *p* < 0.001; ****: *p* < 0.0001.

**Figure 6:**
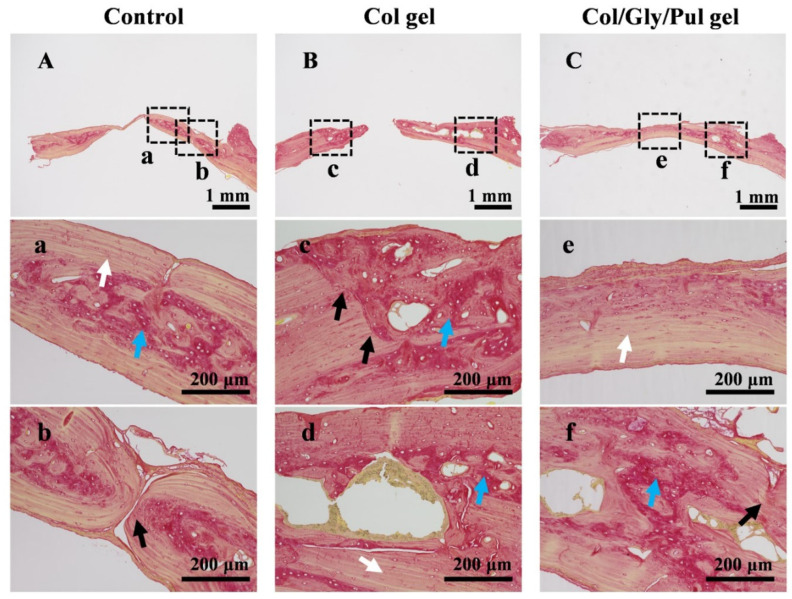
Sirius red stain sections of bone tissues after eight weeks around the (**A,a,b**) Control, (**B,c,d**) Col gel, and (**C,e,f**) Col/Gly/Pul gel. Mature, regular fibrous groups were marked by white arrows; immature, disorganized fibrous tissues marked by blue arrows; and the boundary between the old and new bone marked by black arrows.

Reconstructed three-dimensional micro-computed tomography (micro-CT) pictures of the parietal bones from different samples are displayed in Figure 5B. In the control group, the quantity of new bone was less, and a large defective area was observed in the center. In the Col gel, a notable defective area was observed in the center, and the new bone forming in the marginal part was not completely solid. In the Col/Gly/Pul gel, bone regeneration was the most prominent, and the defective region nearly filled with new bone tissue. The BMD, Tb. N, and BV/TV were the highest and BS/BV and Tb. Sp the lowest in the Col/Gly/Pul gel. The Col gel showed a higher BS/BV than that of the control group (Figure 5C–G). These findings suggest that new bone in the Col/Gly/Pul gel had the highest BMD, level of bone mineralization, and number of new trabeculae, as well as smaller Tb. Sp, a higher volume of new bone per unit area, and more solid new bone. Sirius red stain sections after eight weeks are shown in Figure 6. In the control group, the region of new bone production was smaller, and the boundary between the new and old bone tissue was clearly evident (black arrows in Figure 6b), suggesting that the union of old and new bone was not strong. Fibrous tissue inside the new bone was irregularly deposited (blue arrows in Figure 6a). In the Col gel, the new bone was not completely covered, and more internal cavities were observed (Figure 6B). The border between old and new bone remained (black arrows in Figure 6C). There were more immature collagen fibers, indicating that the new bone was still in the process of forming and undergoing mineralization (blue arrows in Figure 6c,d). In the Col/Gly/Pul gel, osteogenesis was most evident, and the entire defect nearly filled with new bone (Figure 6C). No visible border between the old and preexisting bone was observed, indicating the highest degree of fusion of the new bone with preexisting bone tissue (black arrows in Figure 6f). A considerable quantity of regular, mature fibrous tissue, along with local, immature fibrous tissue, was deposited in the new bone, suggesting that it had a high degree of mineralization (white arrow in Figure 6e).

The real-time PCR results indicated that, compared to the control group and Col gel, the specific expressions of osteogenesis-related genes (ALP, Runx2, and BMP-2) were higher in the Col/Gly/Pul gel. At day 3, the expression of Runx2 on the Col gel was higher than that on the Col/Gly/Pul gel, and we hypothesized that this might be due to the fact that cells on the Col/Gly/Pul gel were still actively proliferating when the rBMSCs underwent osteogenic differentiation, and excessive cell proliferation caused a decrease in differential expression [57]. BMP-2 can interact with integrin β3 and co-control Smad signaling molecules under the control of matrix stiffness and thus regulate cells [58]. In addition, the ALP activity and calcium deposition data suggested that cells on the Col/Gly/Pul gel exhibited high levels of osteogenic differentiation. These results confirm that the Col/Gly/Pul gel has a beneficial impact on early osteogenic differentiation in vitro. This may be caused by the fact that type I collagen in the gel induces the differentiation of BMSCs to osteoblasts through collagen–integrin interactions, as well as exerts a contributing effect on osteoblast-to-osteogenic differentiation and enhances intercellular adhesion [59,60]. Moreover, the Col/Gly/Pul gel, which degrades and releases collagen relatively slowly, promotes osteogenic differentiation in the long term compared to the Col gel, which degrades rapidly. Results of the in vivo experiments also revealed that, after implantation of the Col/Gly/Pul gel, the most extensive and solid new bone growth was observed at the cranial defect, with a less distinct boundary between the old and new bone, and a higher deposition of mature, tightly arranged collagen fibers. Therefore, we speculate that the Col/Gly/Pul gel with injectable, moldable properties can better fit the bone defect area and thus play a role in promoting bone regeneration in the skull.

Biodegradable polymers, such as Pul, can not only play a crucial role as antimicrobial agents but also in the delivery of antimicrobial drugs [61,62]. The stiffness of the material has an effect on the polarization state, function, and migratory mode of macrophages [63]. Therefore, in the future, the potential of whether Col/Gly/Pul gel has antibacterial properties and can affect macrophage polarization should be explored.

In summary, the Col/Gly/Pul gel facilitates the initial attachment, proliferation, and spreading of rBMSCs; increases the expression of osteogenesis-related genes (ALP, Runx2, andBMP-2); and promotes ALP activity and calcium deposition. Its injectability makes it more suitable for the cranial defects of rat, and it promotes the regeneration of bone defects. The newborn bone generated using the Col/Gly/Pul gel was denser and more extensive.

## 3. Conclusions

**Figure 7:**
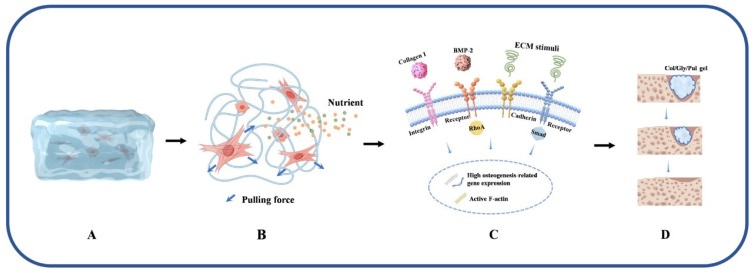
Schematic illustration of the principle of the Col/Gly/Pul gel to promote bone regeneration and repair. (**A**) Col/Gly/Pul gel provides a scaffold for cell growth. (**B**) Col/Gly/Pul gel provides mechanical stimuli to the cells and facilitates the diffusion of nutrients. (**C**) Col/Gly/Pul gel promotes osteogenic differentiation through multiple signaling pathways. (**D**) Regeneration of new bone tissue with degradation of the Col/Gly/Pul gel. This figure was drawn using Figdraw.

Herein, as shown in Figure 7, we modified collagen with Gly and Pul to obtain a Col/Gly/Pul gel with injectability, plasticity, degradation resistance, and better viscoelasticity. In vivo and in vitro results showed that the Col/Gly/Pul gel could promote the adhesion, proliferation, and osteogenic differentiation of rBMSCs on the gel and help bone repair and regeneration of cranial defects in rats, which may be attributed to the different sizes of pores in the Col/Gly/Pul gel, which provide favorable conditions for cell attachment, proliferation, spreading, and nutrient diffusion. The suitable viscoelasticity of the gel promotes osteogenic differentiation. Moreover, the injectability and plasticity of the Col/Gly/Pul gel allow it to better adapt to irregular defects and withstand external loading, thus providing a valuable solution to the clinical challenge of treating localized complex bone defects. Future investigations should be carried out to examine the possible antibacterial properties of the gel and the impact it might have on macrophages.

## 4. Materials and Methods

### 4.1. Preparation of Materials

**Scheme 1:**
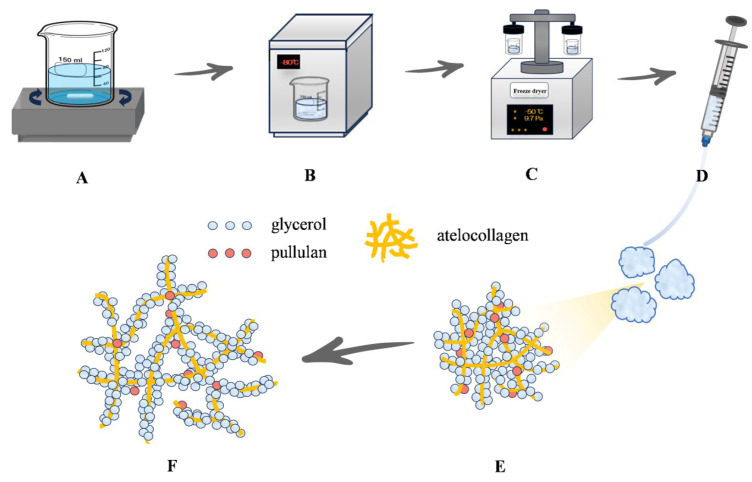
Schematic diagram of the design and preparation of an injectable gel composed of atelocollagen, glycerol, and pullulan (Col/Gly/Pul). (**A**) Col, Gly, and Pul were stirred at room temperature (23–25 °C) for 5 h. (**B**) The mixture was frozen at −80 °C for 30 min. (**C**) The material was dried with a freeze dryer for 12 h. (**D**) Injectable state of the Col/Gly/Pul gel. (**E**) Col/Gly/Pul gel before swelling. (**F**) Network of the swollen Col/Gly/Pul gel.

Glycerol (510 mg; Nacalai Tesque, Inc., Kyoto, Japan), 30 mg Pul (Tokyo Chemical Industry Co., Ltd., Tokyo, Japan), 12 mL IPC-50 Col (bovine hide-derived atelocollagen; Koken Co., Ltd., Tokyo, Japan), and 24 mL of 1 mM NaOH were stirred for 5 h using a magnetic stirrer. After mixing, the materials were frozen at −80 °C for 30 min and thoroughly dried with a freeze dryer (EYELA FDU-2110; Tokyo Rikakikai Co., Ltd., Tokyo, Japan) for approximately 12 h (Scheme 1). The prepared Col/Gly/Pul gel (100 µL/well) was added into a 24-well plate during the subsequent in vitro experiments.

The Col gel was prepared according to the manufacturer’s instructions. The sterile precursor solution was made by mixing the following in order: 1 mL of 10× phosphate-buffered saline (PBS; Nacalai Tesque, Inc., Kyoto, Japan), 0.1 mL of 1 M HEPES (pH 7.4; Nacalai Tesque, Inc., Kyoto, Japan), 0.1 mL of 1 M NaHCO_3_, 0.8 mL distilled water, and 8 mL IPC-50 Col. This solution (300 µL/well) was added into a 24-well plate and placed in an incubator at 37 °C for 2 h to complete gelation.

### 4.2. Characterization of Gels

The inner microstructures of the gels were observed via SEM (S-4800; Hitachi, Tokyo, Japan). FT-IR spectroscopy (IRAffinity-1S; Shimadzu, Kyoto, Japan) was used to analyze bond features of the gels. Viscoelasticity was observed using a rheometer (Rheosol-G5000; UBM, Kyoto, Japan) via amplitude-strain and frequency sweeps at 37 °C. Thixotropic study was observed by viscometer (TV-200; TOKISANGYO, Tokyo, Japan). A 25 mL volume of PBS and 400 mg of Col/Gly/Pul gels were added into 50 mL tubes and placed into a 37 °C thermostat. The gels were collected at predetermined time points (0.5, 1, 2, 3, 4 and 5 h), whereafter surface water was removed, and the gels were weighed. The swelling ratio was calculated using the following equation:

Swelling ratio%=WtWi×100

where *Wt* and *Wi* represent the gel mass at time *t* and the initial gel mass, respectively.

### 4.3. In Vitro Experiments

#### 4.3.1. Cell Culture and Seeding

The rBMSCs were obtained from femurs of eight-week-old Sprague–Dawley (SD) rats (Shimizu Laboratory Supplies Co., Kyoto, Japan) and cultured as previously described [64]. Briefly, rBMSCs were cultured in 75 cm^2^ culture flasks with Eagle’s minimum essential medium (E-MEM) including a 1% antibiotic–antimycotic mixture and 10% fetal bovine serum (FBS) for cell adhesion and morphology. The rBMSCs were also cultured in α-MEM, including a 1% antibiotic–antimycotic mixture, 10% FBS, and supplemented with the osteogenic differentiation-inducing components, 10 nM dexamethasone and 10 mM β-glycerophosphate (FUJIFILM Wako Pure Chemical Co., Osaka, Japan), for osteogenesis-related gene expression, ALP activity, calcium deposition, and Alizarin Red staining. Before cell seeding, the third-generation cells adherent to tissue culture plastic were characterized by multipotent differentiation potential and cluster of differentiation (CD) marker, and then set into a 24-well plate at a density of 6 × 10^4^ cells/well for subsequent in vitro experiments. Except for β-glycerophosphate, the rest of the reagents were sourced from Nacalai Tesque Inc., Kyoto, Japan. The cell culture medium was replaced every three days.

#### 4.3.2. Cell Adhesion and In Vitro Degradation

After 1, 3, and 6 h of seeding, cell adhesion was analyzed by the CellTiter-Blue Cell Viability Assay (Promega, Co., Madison, WI, USA). The samples were washed thrice with PBS and exposed to 250 µL E-MEM and 50 µL Cell Titer-Blue Reagent. After culturing for 2 h, 100 µL of the reagent in each sample was collected and added into a 96-well plate. The absorbance of the solutions was assessed by a microplate reader (SpectraMax M5; Molecular Devices, San Jose, CA, USA) at wavelengths of 560/590 nm. In vitro degradation experiments were performed, according to a previous study [65]. A 25 mL volume of PBS and 2000 mg swollen gels were added into 50 mL tubes and placed into a 37 °C thermostat. In all samples, fresh PBS was added weekly. The gels were collected at predetermined time points (1, 3, 7, 14, 21, and 28 days), whereafter surface water was removed, and the gels were weighed. The remaining gel mass was calculated using the following equation:

Gel mass remaining%=WtWi×100

where *Wt* and *Wi* represent the gel mass at time *t* and the initial gel mass, respectively.

#### 4.3.3. Cell Morphology

After 24 h of culture, the cell morphology of all samples was observed after fluorescent staining. The samples were immobilized by 4% paraformaldehyde solution for 20 min and permeabilized through 0.2% (*v*/*v*) Triton X-100 for 30 min. After rinsing with PBS, the samples were subjected to Blocking One reagent (Nacalai Tesque Inc., Kyoto, Japan) for 30 min and stained for 1 h. The nuclei were labeled by 40,6-diamidino-2-phenylindole (DAPI; AAT Bioquest Inc., Pleasanton, CA, Canada), and actin filaments (F-actin) were labeled by Alexa Fluor 488 phalloidin (Phalloidin; Thermo Fisher Scientific Inc., Tokyo, Japan). Following that, a confocal laser-scanning microscope (LSM700; Carl Zeiss AG, Wetzlar, Germany) was used in order to observe the cells.

#### 4.3.4. Specific Expression of Osteogenesis-Related Gene

The specific expression levels of osteogenesis-related genes were assessed after the cells had been cultured to induce differentiation for 3, 7, or 14 days. To remove the gels and collect the cells, Col and Col/Gly/Pul gel cultures were centrifuged after being treated with 2 mg/mL collagenase type 2 (Sigma-Aldrich, St. Louis, MO, USA) for 30 min [66]. Total RNA of the rBMSCs was collected by RNeasy Mini Kit (Qiagen, Venlo, The Netherlands). Each RNA sample (10 µL) was converted into cDNA via reverse transcription by Prime Script RT kit (TaKaRa Bio Inc., Shiga, Japan). The PCRmax ECO 48 Real-time qPCR System (Cole-Parmer Instrument Co., Tokyo, Japan) was utilized to obtain precise measurements of the ALP and Runx2 expression levels after 3 and 7 days of culture, as well as those of BMP-2 after 14 days of culture. Using the ∆∆Ct approach, the relative expression levels of reactive genes in every group were standardized to the expression level of the housekeeping gene, which was glyceraldehyde 3-phosphate dehydrogenase (GAPDH).

#### 4.3.5. ALP Activity

The cells were collected after being cultivated to induce differentiation for either 7 or 14 days, as stated in Section 4.3.4. Then, 300 µL of 0.2% Triton X-100 was added in each sample and mixed thoroughly to induce cell lysis. Following the manufacturer’s instructions, the ALP pNPP Liquid Substrate from an enzyme-linked immunosorbent assay kit (Sigma-Aldrich, St. Louis, MO, USA) was used to detect the amount of ALP. For the purpose of terminating the reaction, 50 µL of 3 M NaOH was introduced into 200 µL of the reaction substrate. The evaluation of the generation of p-nitrophenol was carried out by utilizing a microplate reader to measure the absorbance of the solution at a wavelength of 405 nm. The amount of DNA was determined with PicoGreen dsDNA Assay Kit (Thermo Fisher Scientific Inc., Tokyo, Japan). The relative amount of ALP was then calculated based on the amount of DNA found in the sample.

#### 4.3.6. Calcium Deposition

After differentiation-inducing cultivation by α-MEM for 21 days, calcium deposits in the ECM of samples were dissolved in 10% formic acid and collected. The Calcium E-Test Kit (FUJIFILM Wako Pure Chemical Co., Osaka, Japan) was used to determine the quantity of calcium in these deposits. The collected medium (50 µL) was mixed with 1 mL calcium emission test reagent and 2 mL kit buffer. The reaction products were then identified using a 96-well microplate reader (SpectraMax M5, San Jose, CA, USA) at a wavelength of 610 nm. The calcium concentration was determined from the absorbance of the relative standard curve.

#### 4.3.7. Alizarin Red Staining

After culturing in α-MEM for 28 days, Alizarin Red staining was performed with the Calcified Nodule Staining kit (Cosmo Bio Co., LTD., Tokyo, Japan). The samples were washed thrice with PBS, 500 µL methanol was then added to them, and they were thereafter fixed at 4 °C for 20 min. Methanol was removed and the samples then washed thrice with purified water, stained with 400 µL stain solution for 5 min, and washed with buffer solution. The samples were observed by all-in-one fluorescence microscope (BZ-X800; Keyence Co., Osaka, Japan).

### 4.4. In Vivo Experiments

#### 4.4.1. The Surgical Procedures of SD Rats

A total of 12 eight-week-old SD rats (male, 180–200 g) were randomly split into three groups. Following general anesthesia and cleaning of the surgical region, a 20 mm sagittal incision was created along the middle of the rat’s head, and the muscle and periosteum separated to expose the parietal bone. A 5 mm defect was created on each side of the sagittal suture using a trephine bur (φ5.0/4.0 mm; IMPLATEX Co., Ltd., Tokyo, Japan) and rinsed with sterile saline. In the Col gel group, a Col gel with a diameter of 5 mm and thickness of 1 mm was placed onto the defect. In the Col/Gly/Pul gel group, 0.2 mL hydrogel was placed onto the defect. The periosteum and muscle were sutured in layers. Buprenorphine and gentamicin were given daily for three days after the operation to reduce postoperative pain and infection.

#### 4.4.2. Micro-CT Analysis and Histology of Sirius Red Stain Sections

After eight weeks of surgical procedure, the rats were euthanized with pentobarbitone. Soon after dissection, the parietal bones were put into saline solution and scanned with a micro-CT system (SkyScan1275; Bruker, Billerica, MA, USA) under the condition of 110 kV and 55 µA with no filter. By using CT reconstruction and morphometric software (NRecon 1.7.4.2, DataViewer 1.5.6.2, CTAn 1.17.7.2; Bruker, Billerica, MA, USA), the bone mineral density (BMD), bone volume-to-total volume (BV/TV), bone surface-to-BV (BS/BV), trabecular number (Tb. N), and Tb. separation (Tb. Sp) of the region of interest, defined as a column surrounding a defect with a diameter of 5 mm and height of 505 µm, were quantified to evaluate bone regeneration. Following the micro-CT scanning, samples were immobilized by 70% ethanol solution and then stained with Sirius red stain to evaluate bone formation. Histomorphometric characteristics of the sections were analyzed using an all-in-one fluorescence microscope (BZ-X800; Keyence Co., Osaka, Japan).

### 4.5. Statistical Analysis

Characterization analyses and in vitro experiments were carried out in triplicate. Statistical analyses were conducted using GraphPad Prism 9.0 software, and the degree of statistical significance assessed via one-way analysis of variance, followed by Bonferroni’s post hoc test. All quantitative results are presented as the mean ± standard deviation, and *p* < 0.05 is considered statistically significant, whereas *p* < 0.01 is considered highly statistically significant.

## 5. Patents

S.K. and Y.T. applied for a patent resulting from the work reported in this manuscript to the Japan Patent Office (application number: 2024-177801).

## Data Availability

All data and materials are available on request from the corresponding author. The data are not publicly available due to ongoing research using a part of the data.
